# A Text-Book on Surgery

**Published:** 1898-04

**Authors:** 


### Book Reviews.

A Text-Book on Surgery.—General, operative, and mechanical, by John A. Wyeth, Professor of Surgery in and President of the Faculty of the New York Polyclinic Medical School and Hospital; late Surgeon to Mount Sinai Hospital and Consulting Surgeon to St. Elizabeth's Hospital, etc., etc., etc. Third edition, revised and enlarged. New York: D. Appleton & Co., 1898.

This work is entitled to great respect, coming from one of the foremost surgeons and teachers in our country, and published by one of the best publishing houses in the United States. The place that "Wyeth's Surgery" already holds in the minds of the profession will be enhanced by the present enlarged edition. Some new illustrations decorate its pages.

As a plain guide in surgical technique, we can indorse it *in toto*, but as a historical or scientific work it lacks some essential points. As an example, we would refer to page 693, where the following statement is found:

"Within recent years the operation of opening into the common gall-duct for obstruction due to calculus, has been considered extra hazardous and difficult, and surgeons have suggested (Nussbaum, 1880) making a fistulous opening between the gall-bladder and the small intestine near the junction of the duodenum and the jejunum. The first operation was done by Monastyrski in 1887, but was unsuccessful. Dr. Jacob Michaux, of Richmond, Va., one of the first to perform this operation in the United States, suggested the operation of cholecysto-enterostomy."

It is difficult to know why the credit due to Drs. Von Winiwarter and J. McFadden Gaston should have been withheld. It is also surprising that the author should have supposed that the position of the operation he mentions should have determined all others near the duodenum and the junction with the jejunum.

The operation which we have been accustomed to regard as the very first ever performed for the junction of the gall-bladder with any portion of the intestinal canal was done by Winiwarter, and was done many years before that done by the author named, *i. e.*, Monastyrski.

The English books have been quite accurate in this respect. Mention has been given by Greig Smith and Mayo Robson of the operation of cholecysto enterostomy. The latter says: "In 1880 Nussbaum suggested the possibility of this operation in cases of obstructive jaundice. (Deutsche Chirurgie, Lieferung, 44, p. 94).

"On the 20th July, 1880, Winiwarter, of Liege, first performed the operation, establishing a communication between the gall bladder and the colon.

"Dr. Gaston, of Atlanta, apparently quite independently of any previous suggestions, in a series of experiments on dogs, showed how a communication might be established between the duodenum and the gall-bladder, which operation he termed duodeno-cholecystotomy, and, as shown by the diagram (Figure 15) that accompanied his second paper (in *Brit. Med. Journal*), this method restores the bile to the intestinal canal, where its physiological functions are accustomed to be performed."

Greig Smith, in his work on Abdominal Surgery, page 627, says under the head of "Entero-Cholecystotomy": "The original operation of Winiwarter, already referred to, successfully established a communication between the gall-bladder and the colon. In this situation, the physiological effects of the biliary secretion were lost. Dr. Gaston, of Atlanta, Ga., in a series of instructive experiments on dogs, showed how a communication might be established between the duodenum and the gall-bladder, thus preserving to the system whatever value the bile may have."

In this country H. O. Marcy, J. B. Murphy, and W. E. B. Davis, have expressed their views of "Gaston's Operation," as called by the late Dr. E. S. Gaillard, in an editorial appearing in the same number of his journal in which appeared Dr. Gaston's first published suggestion of the operation, in October 1884. The *Atlanta Medical and Surgical Journal* for September and October, 1884, were the channels containing the publication of the "Experimental Cholecystotomy." The remark as to Nussbaum by Wyeth, is correct, but the operation of Michaux is not so well established in priority over other American surgeons.

Dr. Wyeth has very full accounts of aneurisms and amputations. His views on pyemia and septicemia are practical, while the remarks on localization of abscesses of the brain seem to us somewhat vague, and uncertain. The advice to operate in these cases of brain suppuration is good, but the method of diagnosis is not positive. We prefer Ashhurt's conservative position in this branch of surgery.

